# Wide-field calcium imaging of cortex-wide activity in awake, head-fixed mice

**DOI:** 10.1016/j.xpro.2021.100973

**Published:** 2021-11-20

**Authors:** Chi Ren, Takaki Komiyama

**Affiliations:** 1 Neurobiology Section, Center for Neural Circuits and Behavior, Department of Neurosciences, and Halıcıoğlu Data Science Institute, University of California San Diego, La Jolla, CA 92093, USA

## Abstract

Characterizing cortex-wide neural activity is essential for understanding large-scale interactions among distributed cortical regions. Here, we describe a protocol using wide-field calcium imaging to monitor the cortex-wide activity in awake, head-fixed mice. This approach provides sufficient signal-to-noise ratio and spatiotemporal resolution to capture large-scale neural activity in behaving mice on a moment-by-moment basis. The use of genetically encoded calcium indicators allows longitudinal imaging over months and can achieve cell-type specificity. We also describe a pipeline to process the imaging data.

For complete details on the use and execution of this protocol, please refer to Makino et al. (2017) and Liu et al. (2021).

## Highlights

• Skull-intact surgical preparation to allow optical access to the mouse dorsal cortex
• Imaging cortex-wide calcium activity in awake, head-fixed mice
• An analysis pipeline to extract neural activity from wide-field image frames

## Graphical abstract

**Figure:**
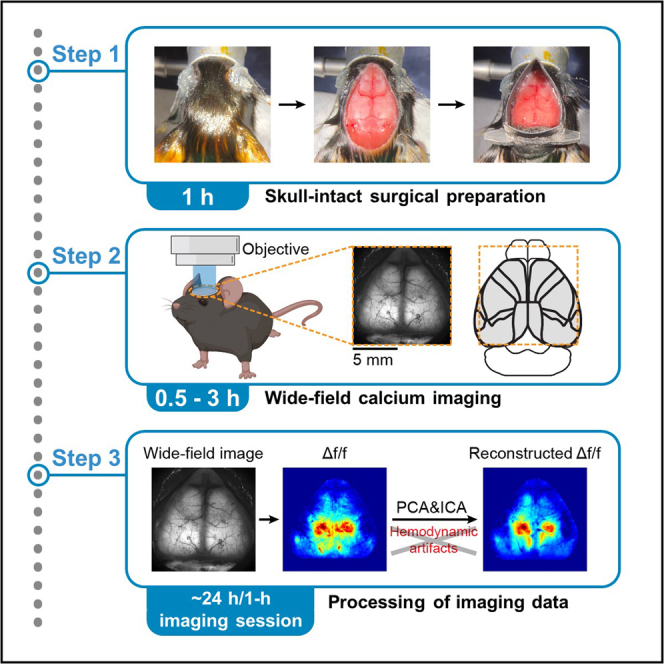

## Before you begin

Wide-field calcium imaging with genetically encoded calcium indicators, especially the GCaMP family, has become an attractive approach to visualize the cortex-wide neural activity in living mice under various sensory and cognitive processes (Cardin et al., 2020; Ren and Komiyama, 2021). It provides a good spatiotemporal resolution by simultaneous illumination of large cortical areas and a high signal-to-noise ratio from bright GCaMP fluorescence. Genetic encoding of GCaMP also enables stable expression for longitudinal recordings and probing specific neuronal populations through genetically restricted expression. The protocol below describes the specific steps for using wide-field calcium imaging to monitor the cortex-wide activity in transgenic mice expressing GCaMP6s widely in cortical excitatory neurons. However, this protocol can also be used to image the cortex-wide activity in specific neuronal populations, such as neurons in specific cortical layers (Gilad et al., 2018; Valley et al., 2020) and different subtypes of cortical inhibitory neurons (Allen et al., 2017). Furthermore, this protocol can be adapted to image the cortex-wide fluorescence signals from other genetically encoded indicators, such as indicators for neurotransmitters (Lohani et al., 2020; Xie et al., 2016). In addition, this protocol can also be incorporated with other recording modalities, such as electrophysiological recordings, to simultaneously record from the cortex and deep brain regions (Liu et al., 2021; Peters et al., 2021). Therefore, this protocol has a broad application to study different macroscopic properties of brain functions. The readers are encouraged to reference a recent protocol paper of similar experiments (Couto et al., 2021). It should be noted that this protocol, together with any modifications, should be approved by the Institutional Animal Care and Use Committee (IACUC) before implementation.

### Set up mouse crosses for broad expression of GCaMP6s in cortical excitatory neurons

**Timing: ∼3 months**

1. Cross tetO-GCaMP6s transgenic mice (Wekselblatt et al., 2016) to CaMKIIα-tTA transgenic mice (Mayford et al., 1996) to generate offspring with GCaMP6s widely expressed in cortical excitatory neurons. Both homozygotes and heterozygotes can be used as parents for crossing. When heterozygous parents are used, offspring carrying both transgenic alleles should be identified by PCR genotyping. GCaMP6s expression can also be confirmed by fluorescence imaging through the intact skull in an early step of the surgical procedure.

***Note:*** The use of transgenic mice with endogenous expression of the indicator allows broad and stable expression in specific neuronal populations throughout the cortex. The exact transgenic mouse lines depend on the purpose of the study and the neurons of interest. If particular transgenic lines are not available, expression of the indicator throughout the cortex can also be achieved using AAV-PHP.eB. This AAV variant crosses the blood-brain barrier and delivers genes systemically in the brain (Chan et al., 2017). Although the authors do not have direct experience using this approach for the protocol described here, see reference (Michelson et al., 2019) for a detailed comparison between transgenic and AAV-PHP.eB-mediated expression of GCaMP6s in wide-field calcium imaging.

***Note:*** The exact time varies depending on the age of mice required for the experiments. For example, we usually start the surgical preparation on ∼2–3-month old mice.

### Preparation for skull-intact surgery

**Timing: 10 min**

**Figure 1:**
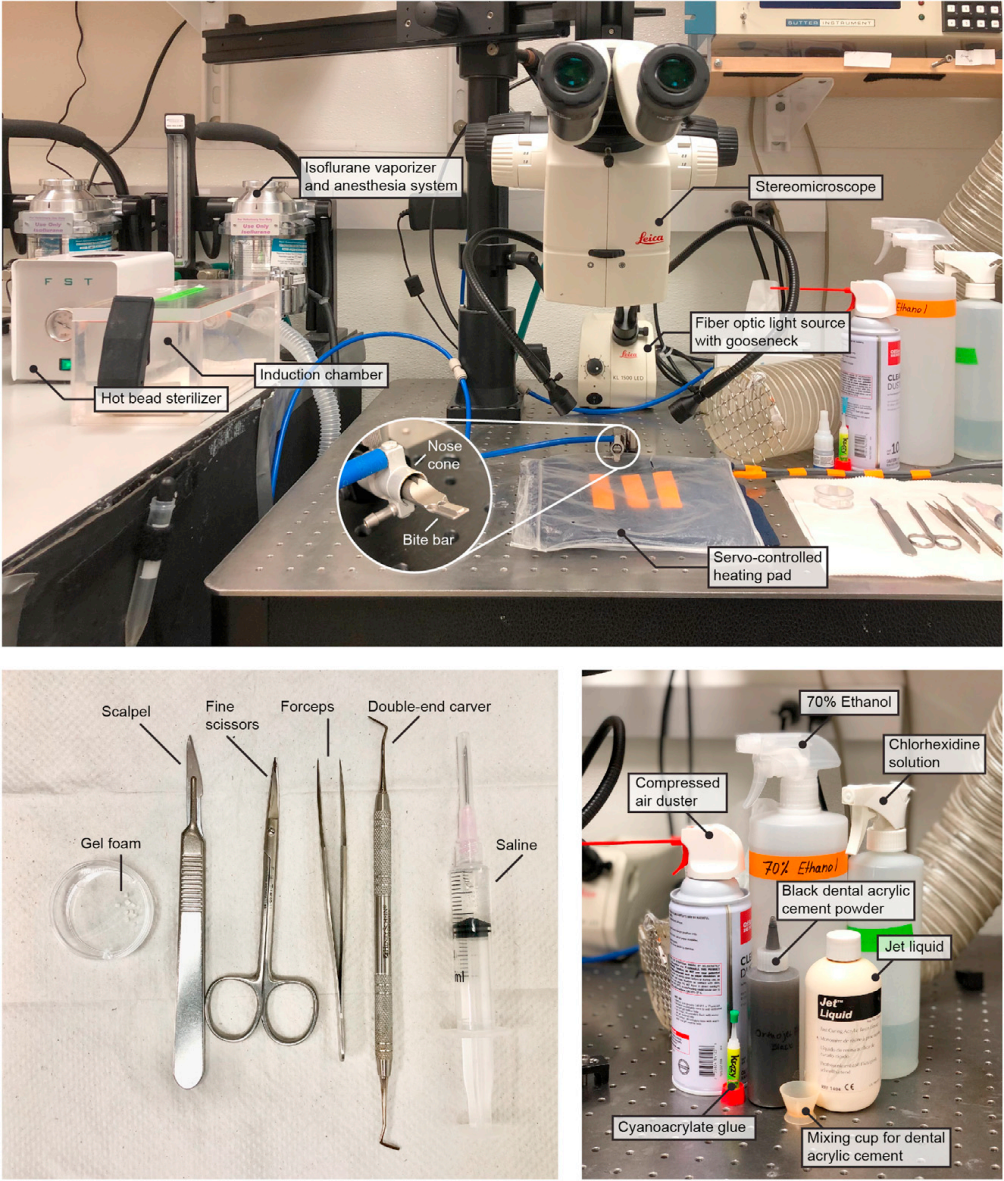
Surgery setup and key reagents for the skull-intact surgery

**Figure 2:**
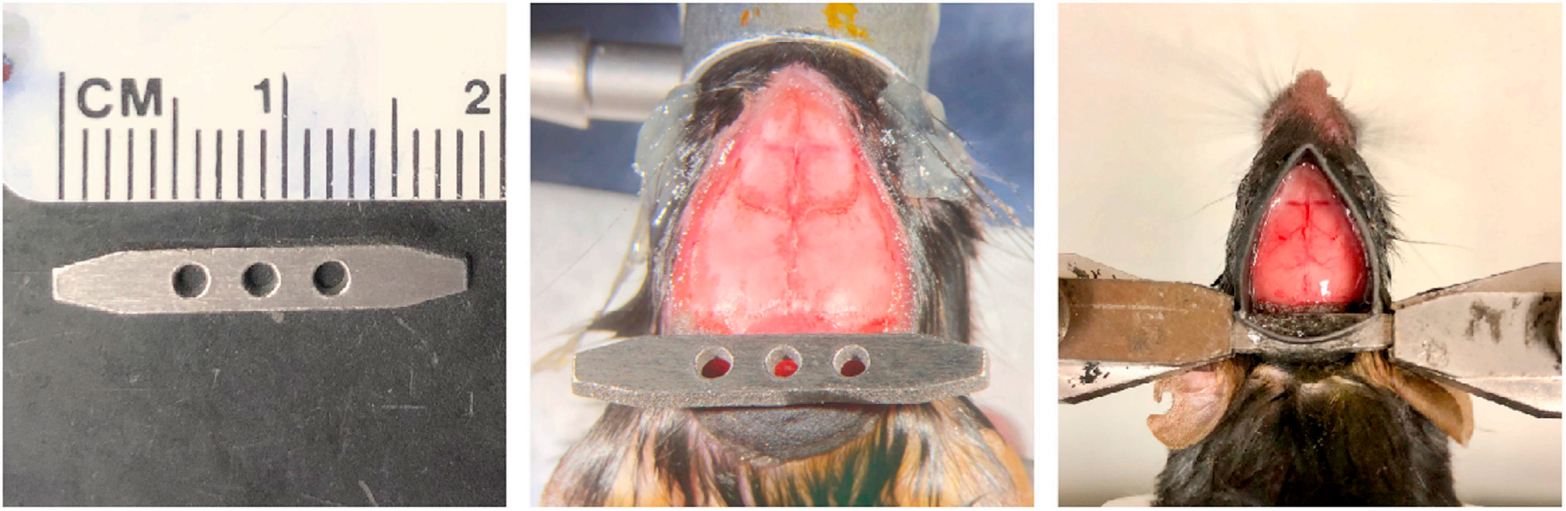
The custom-built stainless steel headbar (left) attached to the skull (middle) for head fixation on the stage (right) under the wide-field microscope

The surgery setup (Figure 1) consists of a stereomicroscope (Leica M125, Leica Microsystems), a fiber optic light source with gooseneck (KL 1500 LED, Leica Microsystems), a servo-controlled heating pad (Harvard Apparatus), a gas anesthesia head holder (nose cone and bite bar, KOPF) and an induction chamber (Vetequip) connected to the isoflurane vaporizer and anesthesia system (Vetequip), a hot bead sterilizer (Fine Science Tools), and surgical tools (Fine Science Tools). In this protocol, we use a custom-built stainless steel headbar attached to the skull for head fixation on the stage under the wide-field microscope (Figure 2). The headbar is custom manufactured (e.g., emachineshop.com). Other designs could also be used if the head-fixation can be achieved without blocking the field of view over the dorsal cortex.

2. Sterilize the surgery table, surgical tools, and headbar.
  a. Sterilize the surface of the surgery table with chlorhexidine solution.
  b. Sterilize surgical tools, including scalpel, scissors, forceps, double-end carver, and the headbar using a hot bead sterilizer.
3. Cut sterile gel foam into ∼ 1 × 1 mm small pieces.

***Note:*** The gel foam (1975, Ethicon) listed here can be used dry or saturated with saline. However, some gel foams must be moisturized with saline before applying to the wound. Check the direction of your gel foam and soak small pieces of gel foam in saline if necessary.

4. Turn on the servo-controlled heating pad which maintains the mouse body temperature at 35°C–37°C during surgery.

## Key resources table

**Table:** 

REAGENT or RESOURCE	SOURCE	IDENTIFIER
**Chemicals, peptides, and recombinant proteins**		

Isoflurane	VetOne	Cat#502017
Chlorhexidine solution (2%)	AgriLabs	NDC 57561-142-03
Saline (0.9% sodium chloride)	ICU Medical	NDC 0990-7983-09
Vaseline Healing Jelly Original	Vaseline	https://www.vaseline.com/us/en/products/healing-jelly/vaseline-healing-jelly-original.html
Betadine (Solimo First Aid Antiseptic, 10%)	Solimo	https://www.amazon.com/Amazon-Brand-Antiseptic-Povidone-Solution/dp/B07CJXW5GY
Cyanoacrylate glue (All Purpose, Precision Tip)	Krazy Glue	https://www.amazon.com/Krazy-Glue-Tube-Original-07/dp/B000Q3GW3A/ref=sr_1_18?crid=3CVVKM5JKHFB8&dchild=1&keywords=krazy+glue&qid=1626673121&sprefix=krazy%2Caps%2C147&sr=8-18
Dental acrylic cement (Contemporary Ortho-Jet™ Powder and Jet Liquid)	Lang Dental	https://www.langdental.com/products-Contemporary-Ortho-Jettm-Powder-50 https://www.langdental.com/products-Jet-Liquid-46
Hydrogen peroxide (3%)	Swan	NDC 0869-0871-43
Buprenorphine Hydrochloride (0.3mg/mL)	Par Pharmaceutical	NDC 42023-179-05
Baytril (100 mg/mL)	Bayer	NADA 141-068
Dexamethasone (2 mg/mL)	VetOne	Cat#501012

**Experimental models: Organisms/strains**		

Mouse: CaMKIIa-tTA, B6; CBA-Tg(Camk2a-tTA)1Mmay/J	The Jackson Laboratory	RRID: IMSR_JAX: 003010
Mouse: tetO-GCaMP6s, B6; DBA-Tg(tetO-GCaMP6s)2Niell/J	The Jackson Laboratory	RRID: IMSR_JAX: 024742

**Software and algorithms**		

HCImage Live	Hamamatsu Photonics	RRID: SCR_015041
MATLAB	MathWorks	RRID: SCR_001622
JADER	Cardoso (1999)	https://www.mathworks.com/matlabcentral/mlc-downloads/downloads/submissions/67527/versions/3/previews/jadeR.m/index.html
Custom MATLAB code for data processing	Makino et al. (2017)	GitHub: https://github.com/CRen2333/Wide-field-calcium-imaging

**Other**		

Leica M125 stereomicroscope	Leica Microsystems	https://www.leica-microsystems.com/products/stereo-microscopes-macroscopes/p/leica-m125-c/
Fiber optic light source with gooseneck (KL 1500 LED)	Leica Microsystems	N/A
Mouse gas anesthesia head holder (nose cone and bite bar)	KOPF	Cat#1923-B
Stainless steel headbar	Custom-made from eMachineShop	N/A
Dental drill handpiece	Midwest Tradition	N/A
Drill bit (FG 1/4)	Henry Schein	Cat#1007205
Scalpel (#3)	Fine Science Tools	Cat#10003-12
Fine scissors	Fine Science Tools	Cat#14060-11
Forceps	Fine Science Tools	Cat#11223-20
Double-end carver	Henry Schein	Cat#101-0333
Compressed air duster	Office Depot	Cat#337994
Gel foam	Ethicon	Cat#1975
Heat shrink tube	Amazon	N/A
Servo-controlled heating pad	Harvard Apparatus	Cat#50-7220F
Hot bead sterilizer	Fine Science Tools	Cat#18000-45
Isoflurane vaporizer and anesthesia system	Vetequip	Cat#901806
Induction chamber for gas anesthesia	Vetequip	Cat#941444
Axio Zoom.V16, objective lens (1 × , 0.25 NA, FWD 56 mm)	ZEISS	RRID: SCR_016980
Illuminator HXP 200C	ZEISS	Cat#423612-9901-000
ORCA-Flash4.0	Hamamatsu Photonics	https://www.hamamatsu.com/jp/en/product/type/C13440-20CU/index.html

## Materials and equipment

This protocol uses a commercial fluorescence stereoscope (Axio Zoom.V16, Zeiss) equipped with a scientific camera (ORCA-Flash4.0, Hamamatsu) for wide-field calcium imaging.

***Alternatives:*** There are a number of vendors that produce wide-field microscopes. Wide-field microscopes can also be custom-built (Couto et al., 2021). Scientific cameras with a large field of view, high quantum efficiency, and fast frame rates are suitable for this protocol.

## Step-by-step method details

### Skull-intact surgical preparation

**Timing: 1 h**

This section describes the steps of a skull-intact surgical preparation that allows longitudinal optical access through the intact skull over months.

1. Anesthetize the adult mouse (between 6 weeks and 4 months old, either gender) with isoflurane (3% in 100% O_2_ for induction) using inhalation in an induction box.
2. Transfer the anesthetized mouse to an isoflurane ventilator with a nose cone. The percent isoflurane is regulated to achieve steady breathing rates (∼1 breath/s) throughout the surgery (usually 1–1.5% in 100% O_2_). The mouse is kept on a servo-controlled heating pad to maintain body temperature, and the breathing rate is monitored during the entire surgery.
3. Cover the eyes with copious amounts of Vaseline (Figure 3A, indicated by white arrowheads) to protect against drying and potential exposure to irritant chemicals (e.g., cyanoacrylate glue and dental acrylic cement) in the following steps.

**Figure 3:**
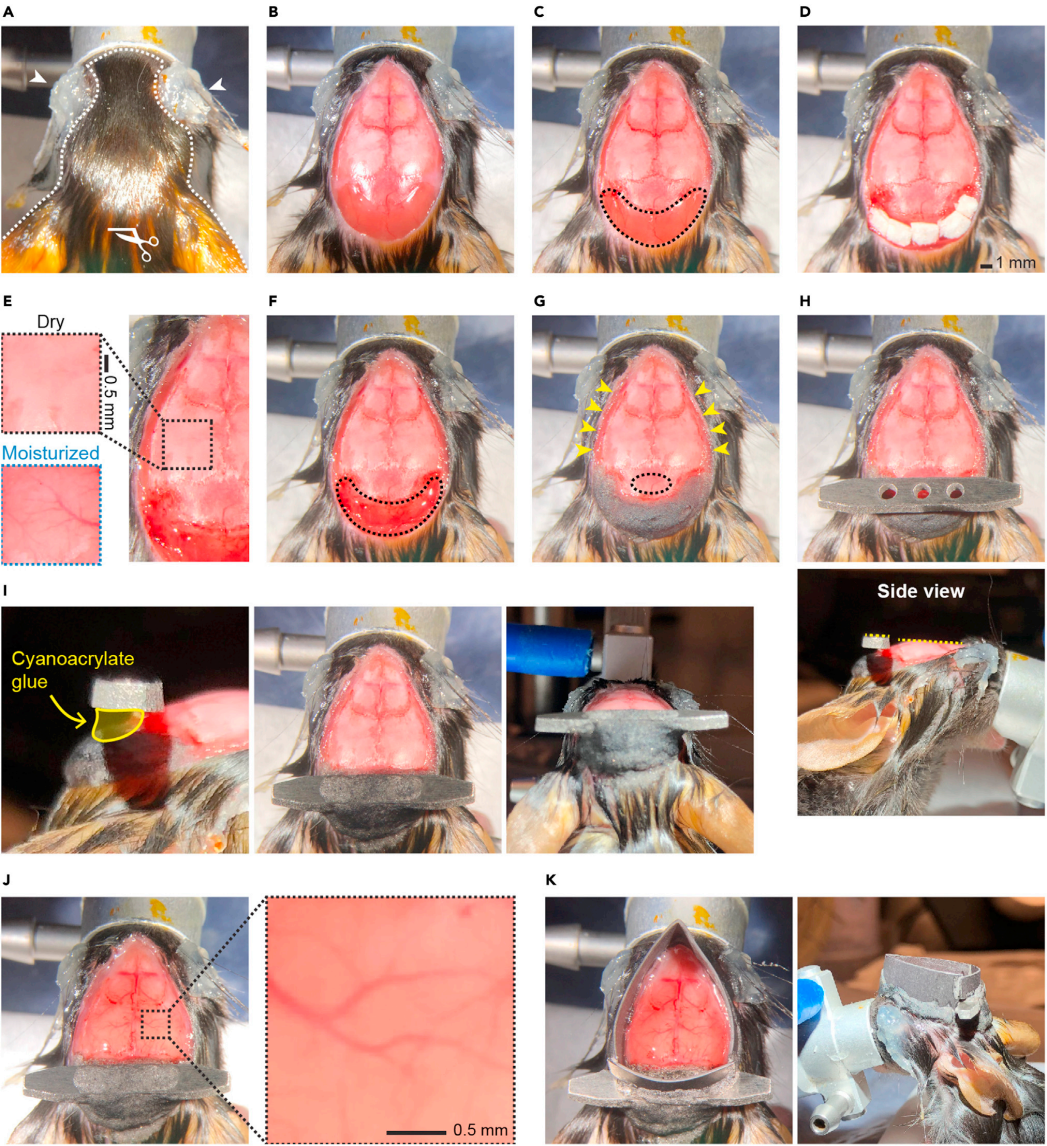
Surgical procedures of the skull-intact preparation for wide-field calcium imaging (A) Protect the eyes with copious amounts of Vaseline (indicated by white arrowheads). Clean and antisepticise the surgical region (marked by the white dashed line). Make a small incision (∼2 mm) on the scalp using fine scissors (marked by the white scissor symbol). (B) Starting from the small incision, cut off a circular piece of the scalp to expose the skull covering the dorsal cortex and cerebellum. (C) Gently remove the periosteum on the skull surface using a scalpel. Note that the skull is less glossy after removing the periosteum compared to (B). Next, detach the muscles from the occipital bone (within the black dashed line) with a scalpel and push the muscles away. (D) Insert small pieces of gel foam into the gaps between muscles and the occipital bone to stop bleeding. Wipe the skull with cotton swabs soaked in 3% hydrogen peroxide to remove any remaining soft tissues. (E) Comparison between moisturized and dry skulls. Note that the dry skull is whiter and less transparent. It is critical to dry the skull completely before applying cyanoacrylate glue, as the glue becomes opaque when encountering a wet surface. (F) Remove the gel foam and apply small drops of cyanoacrylate glue to cover the muscles and the occipital bone (marked by the black dashed line). Let the cyanoacrylate glue cure. (G) Add drops of dental acrylic cement with a double-end carver to build a thick layer on top of the glue. Let the dental acrylic cement cure. Apply cyanoacrylate glue with a double-end carver to cover the skin along the cuts (indicated by yellow arrowheads). (H) Implant the headbar. Apply a small amount of cyanoacrylate glue to the interparietal bone (marked by the black dashed ellipse in G) and place the headbar ∼0.5–1 mm posterior to the lambda. Adjust the angle of the headbar by eyeballing until it is parallel to the transverse plane of the skull (the top side of the headbar and the transverse plane of the skull are marked by yellow dashed lines in the bottom panel). Hold the headbar in place until it stays steady. (I) Secure the headbar. Left, add drops of cyanoacrylate glue to build a glue layer between the headbar and cement (indicated by the yellow shading area). Let the glue cure. Middle and right, apply dental acrylic cement around the headbar to build up the attachment between bones and the headbar. Make sure the bones posterior to the lambda and the middle part of the headbar are entirely covered. Let the dental acrylic cement cure. (J) Build the cyanoacrylate glue layer on the skull. Apply a small amount of cyanoacrylate glue and evenly distribute it to form a thin layer covering the entire skull. Wait for the cyanoacrylate glue to cure to form a solid, smooth, and transparent layer. Repeat 3–4 times to thicken the glue layer. The skull should gradually become more transparent within 20 min, and the blood vessels should become clearly visible compared to the dry skull in (E). (K) Protect the eyes from the excitation light of imaging. Cut a chunk (∼5 mm) of black heat shrink tube (∼2 cm diameter) and glue it to the circumference of the skull using cyanoacrylate glue. Then fill the gap between the heat shrink tube and mouse head with black dental acrylic cement from the outside. Scale bars in (D), 1 mm, in (E and J), 0.5 mm.
4. Clean and antisepticise the scalp and the surrounding region (Figure 3A, marked by the white dashed line).
  a. Wipe the surgical region with cotton swabs soaked in 70% ethanol 2–3 times.
  b. Wipe the surgical region with cotton swabs soaked in betadine until the entire surgical region has been infiltrated.
5. Make a small incision (∼2 mm) on the scalp between two ears using fine scissors (Figure 3A, marked by the white scissor symbol). Starting from the small incision, cut off a circular piece of the scalp to expose the skull covering the dorsal cortex and cerebellum. Retract the remaining scalp around the cuts with forceps to fully expose the dorsal skull (Figure 3B).

**CRITICAL:** We have found that shaving hair or not before cutting off the scalp does not impact the surgical success rate. However, if there is interfering hair left on the skull after cutting off the scalp, remove the hair with clean cotton swabs or forceps to prevent hindrance to a clear field of view for wide-field calcium imaging.

**CRITICAL:** When cutting the scalp close to the eyes, use forceps to lift the scalp and confirm the location of the retro-orbital sinus first. Do not damage the retro-orbital sinus.

6. Gently remove the periosteum on the skull surface using a scalpel. Note that the skull is less glossy after removing the periosteum (Figure 3C, compare with Figure 3B). If bone bleeding happens, use a dry cotton swab to press the bleeding spot until bleeding stops (usually within 5 min). If the bleeding continues after pressing for more than 10 min, terminate the surgery and exclude the animal from the experiment. Rinse the skull with saline several times to remove any bloodstains. Troubleshooting 1

**CRITICAL:** Be particularly gentle when cleaning the periosteum along the bone sutures to avoid bleeding under the skull.

7. Detach the muscles from the occipital bone (Figure 3C, muscles within the black dashed line) with a scalpel and push the muscles away. This is to extend the bone interface for cyanoacrylate glue and dental acrylic cement. This is critical for the steady implantation of the headbar. Next, insert small pieces of gel foam into the gaps between muscles and the occipital bone to stop bleeding (Figure 3D).

***Note:*** Some gel foams must be moisturized with saline before applying to the wound. Check the direction of your gel foam and soak small pieces of gel foam in saline if necessary. Though the gel foam (1975, Ethicon) listed in this protocol can be used both dry and saturated with saline, we note that dry gel form is more efficient in stopping muscle bleedings.

**CRITICAL:** Keeping the skull clean and smooth is critical to achieving even optical access through the dorsal cortex. When detaching the muscles, be gentle to avoid making deep scratches or notches on the occipital bone and skull, which can cause bone bleeding and roughen the skull surface. If bleeding is too heavy to stop with gel foam, press on the bleeding site with clean and dry cotton swabs, and the bleeding usually stops within 5 min. If any blood spreads to the skull and generates stains, rinse the skull with saline and wipe with dry cotton swabs after bleeding stops to remove bloodstains. If bleeding continues for more than 20 min and cannot be slowed down, terminate the surgery and exclude the animal from the experiment.

8. Wipe the skull to remove any remaining soft tissues with cotton swabs soaked in 3% hydrogen peroxide.
9. Wait ∼5 min to dry the dorsal skull. The skull should become whiter and less transparent as it dries (see Figure 3E for the comparison between dry and moisturized skulls). Troubleshooting 2

**CRITICAL:** Cyanoacrylate glue becomes opaque when encountering a wet surface. Make sure the skull is dry before continuing. If the drying is too slow, gently blow the skull with a compressed air duster to facilitate drying and ensure dryness. Do not blow too hard, as a strong airflow may detach the skin around the cuts and cause bleeding near the retro-orbital sinus.

10. Fill the gap between muscles and the occipital bone.
  a. Gently remove the gel foam between muscles and the occipital bone with forceps.
  b. Apply small drops of cyanoacrylate glue to cover the muscles and the occipital bone (Figure 3F, the area encompassed by the black dashed line. Note that the area shows high gloss when covered with cyanoacrylate glue).
  c. After the cyanoacrylate glue cures, add drops of dental acrylic cement with a double-end carver to build a thick layer on top of the glue (Figure 3G).
  d. Apply cyanoacrylate glue with a double-end carver to cover the skin along the cuts (Figure 3G, indicated by yellow arrowheads). Troubleshooting 3
11. Implant the headbar.
  a. Apply a small amount of cyanoacrylate glue to the interparietal bone (Figure 3G, marked by the black dashed ellipse).
  b. Place the headbar ∼0.5–1 mm posterior to the lambda.
  c. Adjust the angle of the headbar by eyeballing until it is parallel to the transverse plane of the skull (Figure 3H, the top side of the headbar and the transverse plane of the skull are marked by yellow dashed lines).
  d. Hold the headbar in place until it stays steady.

**CRITICAL:** Placing the headbar parallel to the transverse plane of the skull is important to reduce distortion in imaging and help alignment across mice. It also keeps the mice in a more comfortable posture during imaging.

12. Secure the headbar.
  a. Add drops of cyanoacrylate glue through the holes on the headbar to build a glue layer between the headbar and cement (Figure 3I, left, indicated by the yellow shading area).
  b. After the glue cures, apply dental acrylic cement with a double-end carver around the headbar to build up the attachment between bones and the headbar. Make sure the bones posterior to the lambda are entirely covered. Embed the middle part of the headbar in cement to secure the headbar further (Figure 3I, middle and right). Troubleshooting 4
  c. Let the dental acrylic cement cure.

**CRITICAL:** Applying enough cyanoacrylate glue and dental acrylic cement is important to firmly fix the headbar onto the skull in living mice due to the small interface between the headbar and the skull. Otherwise, the headbar may detach from the skull during imaging.

13. Build a thin cyanoacrylate glue layer on the skull.
  a. Apply a small amount of cyanoacrylate glue to the skull over the dorsal cortex and evenly distribute it using a double-end carver to form a thin layer covering the entire skull.
  b. Wait for the cyanoacrylate glue to cure to form a solid, smooth, and transparent layer. Curing usually takes ∼5 min.
14. Repeat step 13 3–4 times. The skull should gradually become more transparent within 20 min, and the brain blood vessels should become clearly visible (Figure 3J, compare with the dry skull in Figure 3E). Troubleshooting 5

**CRITICAL:** Applying small amounts of glue multiple times is more efficient than applying a large amount at once as the curing requires moisture in the air.

**CRITICAL:** If bleeding starts after applying cyanoacrylate glue and spreads, carefully drill off the glue with a dental drill (Midwest Tradition) after the glue layer cures. To avoid overheating during drilling, stop every 30 s to let the surface cool down and blow with a compressed air duster to facilitate cooling. Next, blow off the debris with a compressed air duster and reapply cyanoacrylate glue to form a smooth and transparent layer.

15. Cut a chunk (∼5 mm) of black heat shrink tube (∼2 cm diameter) and glue it to the circumference of the skull using cyanoacrylate glue. Then fill the gap between the heat shrink tube and mouse head with black dental acrylic cement from the outside (Figure 3K). This is to minimize the entry of the excitation light (blue light, ∼485 nm) to the eyes during imaging.

***Note:*** There are different colors of the dental acrylic cement powder, but the black one is most effective in blocking the light. If you do not have black dental acrylic cement powder in hand, use the least transparent powder you have. After the cement cures, cover it with a layer of black electrical tape or paint it with black marker pens to help block the light.

**CRITICAL:** Do not let black dental acrylic cement flow onto the transparent glue layer on the dorsal skull to avoid blocking the imaging field of view.

**CRITICAL:** Exposure to the excitation blue light for wide-field calcium imaging can cause retinal damage, especially after frequent imaging or a single long imaging session (e.g., 3 h) (Nakamura et al., 2018). In addition, when imaging is performed in alternating blocks (e.g., 5-min on and 5-min off), the light onset may activate the visual cortex and complicate the interpretation of activity. Therefore, it is critical to minimize the entry of the excitation blue light to the eyes.

16. Inject Buprenorphine for pain relief (0.1 mg/kg of body weight) and Baytril to prevent infection (10 mg/kg of body weight) subcutaneously.
17. After the dental acrylic cement in step 15 is cured, take the mouse off from the nose cone and monitor it as it recovers from anesthesia.
18. Check the optical clarity of the skull-intact preparation frequently after the surgery. The window should stay clear for months when no infection occurs. In our experience, the preparation of most animals can stay clear for at least 2 months. The longest experiment we have performed lasted 10 weeks and the preparation stayed clear throughout the experiment.

**CRITICAL:** Although rare, infection can still happen within the first week after surgery. If the preparation appears cloudy and yellow due to infection in the skull, inject a mixture of Baytril (10 mg/kg of body weight) and Dexamethasone (1 mg/kg of body weight) subcutaneously for consecutive 3–5 days to stop infection and reduce inflammation. If the infection continues or the preparation does not become clear for imaging, terminate the experiment of this animal and exclude it from the study.

### Wide-field calcium imaging in living mice

**Timing: 30 min to 3 h**

This section describes the steps of wide-field calcium imaging through the skull-intact preparation in living mice.

19. Prepare the imaging setup.
  a. Turn on the imaging light source (HXP 200 C, Zeiss), the microscope (Axio Zoom.V16 equipped with an objective lens (1 ×, 0.25 NA, FWD 56 mm), Zeiss), and the camera (ORCA-Flash4.0, Hamamatsu). The filter set for imaging GCaMP signals is commercially installed in the microscope. It consists of a bandpass filter for the excitation light (485 ± 17 nm), a beamsplitter (500 nm), and a tunable bandpass filter centered at 520 nm for the emission light.
  b. Open the HCImage Live software (Hamamatsu). Set frame size to 512 × 512 pixels and frame rate to 30 Hz.

***Note:*** The pixel number and frame rate vary depending on the experimental design and the kinetics of the indicator. For imaging GCaMP signals, frame size is often between 512 × 512 to 1024 × 1024 pixels, and frame rate is in a range of 15–60 Hz.

**Figure 4:**
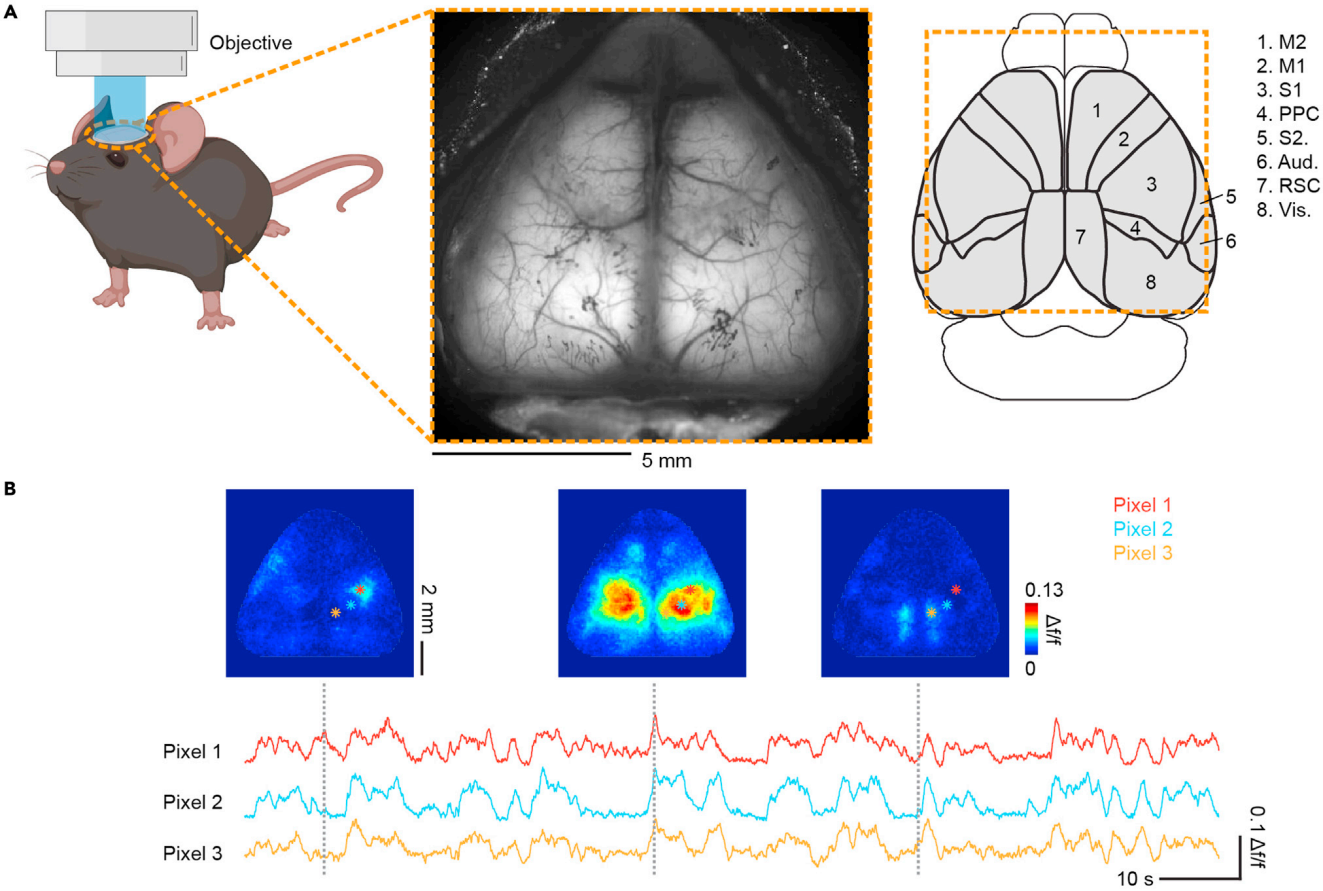
Wide-field calcium imaging in the transgenic mouse with a broad expression of GCaMP6s in cortical excitatory neurons (A) Left, imaging setup. Middle, an example field of view showing the fluorescence signal from most of the dorsal cortex. Note that the lateral blood vessels around the edges are out of focus and appear blurry due to the curvature of the cortex. Right, cortical regions (based on the mouse brain atlas from the Allen Institute (Wang et al., 2020)) simultaneously recorded by wide-field calcium imaging. Orange dashed box represents the area within the FOV. M2, Secondary motor cortex; M1, primary motor cortex; S1, primary somatosensory cortex; PPC, posterior parietal cortex; S2, secondary somatosensory cortex; Aud., auditory cortex; RSC, retrosplenial cortex; Vis., visual cortex. (B) Example image frames of cortex-wide activity (Δf/f) showing different patterns and activity traces of individual pixels in a behaving mouse. Not that the activation in small cortical areas can be resolved by wide-field calcium imaging. Gray dashed lines indicate the time of image frames in activity traces of example pixels. Adapted from Ren and Komiyama (2021). Scale bars in (A), 5 mm, in (B), for image frames, 2 mm, for activity traces, veritical, 0.1 Δf/f, horizontal, 10 s.

20. Prepare the mouse for imaging.
  a. Fix the mouse to the stage under the microscope through the headbar.
  b. Clean the window surface with cotton swabs soaked in 70% ethanol.
21. Choose the field of view (FOV).
  a. Adjust the position of the stage to place the cortex in the center of the FOV (∼11 mm × 11 mm).
  b. Adjust the height of the objective until the blood vessels are in focus. Due to the curvature of the cortex, the lateral blood vessels around the edges will be out of focus and appear blurry (Figure 4A, middle).

**CRITICAL:** Minimize the power of excitation light during this process to avoid photobleaching of GCaMP signals.

22. Adjust the power of the excitation light (blue light, ∼485 nm for imaging GCaMP signals). To avoid saturation, the brightest pixels (usually located in the retrosplenial cortex) are usually around 50–60% of the full dynamic range. For imaging GCaMP6s in cortical excitatory neurons, the light power is ∼10–20 mW to achieve a good signal quality without inducing significant photobleaching.

***Optional:*** During repeated imaging, we use the average frame from the first imaging session as a reference and align the FOV in the following imaging sessions to this reference image based on landmarks (e.g., blood vessels, small bright spots on dental acrylic cement) through eyeballing. The x–y location can be aligned by adjusting the position of the stage as in step 21. No precise x–y alignment is required during image acquisition, as small misalignments will be corrected by the offline image registration (see ‘image registration across sessions within the same mouse’ section for details). The z depth can be aligned by adjusting the height of the objective until the blood vessels show similar clarity as the reference image.

***Note:*** With a power of 10–20 mW, the imaging depth is down to ∼200 μm, covering cortical layer 1 and shallow layer 2/3. As the light intensity decays quickly during the propagation in brain tissue (decreasing to 50% at ∼100 μm and 10% at ∼200 μm) (Yizhar et al., 2011), the majority of signals likely come from layer 1.

***Note:*** To minimize potential photobleaching due to repeated imaging in longitudinal studies, imaging can be performed with a certain duty cycle and increase intervals between imaging sessions. Here we provide our imaging schedule as a reference for repeated imaging: imaging is performed with a 50% duty cycle (5-min on and 5-min off) for ∼30 min every other day for a 22-day behavior task (11 imaging days in total).

23. Set the acquisition to internal or external trigger mode in HCImage Live software based on the experiments and start imaging acquisition.

***Note:*** Under the internal mode, the image acquisition is controlled by HCImage Live software and independent of external triggers. The internal mode is used when no alignment or synchronization is required between imaging and other recordings, such as imaging spontaneous cortical activity.

***Note:*** Under the external trigger mode, the image acquisition is controlled by external devices through TTL. It is suitable when imaging needs to be synchronized to behavior recordings or other recording modalities, such as imaging cortex-wide activity during task performance or simultaneous recording with electrophysiology.

## Expected outcomes

Using this protocol, we can perform a skull-intact surgical preparation that allows optical access to most of the dorsal cortex for imaging cortex-wide neural activity in living mice (Figure 4A). The surgery is less invasive than craniotomy so the mice can recover from the surgery quickly and have a lower infection probability. According to our experience, the skull-intact preparation can stay clear for at least 2 months, and no obvious photobleaching occurs with appropriate intensity of the excitation light, allowing repeated imaging over time. This is particularly beneficial for studies requiring longitudinal recordings, such as monitoring the cortex-wide activity throughout long-term learning. Wide-field calcium imaging also provides a good spatiotemporal resolution and a high signal-to-noise ratio to resolve diverse cortex-wide activity patterns on a moment-by-moment basis (Figure 4B). Taken together, these advantages have made wide-field calcium imaging an attractive tool to uncover cortex-wide dynamics in various cognitive processes, such as sensory perception, sensorimotor integration, decision-making, and learning (Cardin et al., 2020; Ren and Komiyama, 2021). Here, we use two of our studies as example cases to demonstrate the capacity of wide-field calcium imaging in resolving cortex-wide dynamics in living mice.

### Case 1: Characterizing learning-related dynamics of cortex-wide activity during motor learning (Makino et al., 2017)

**Figure 5:**
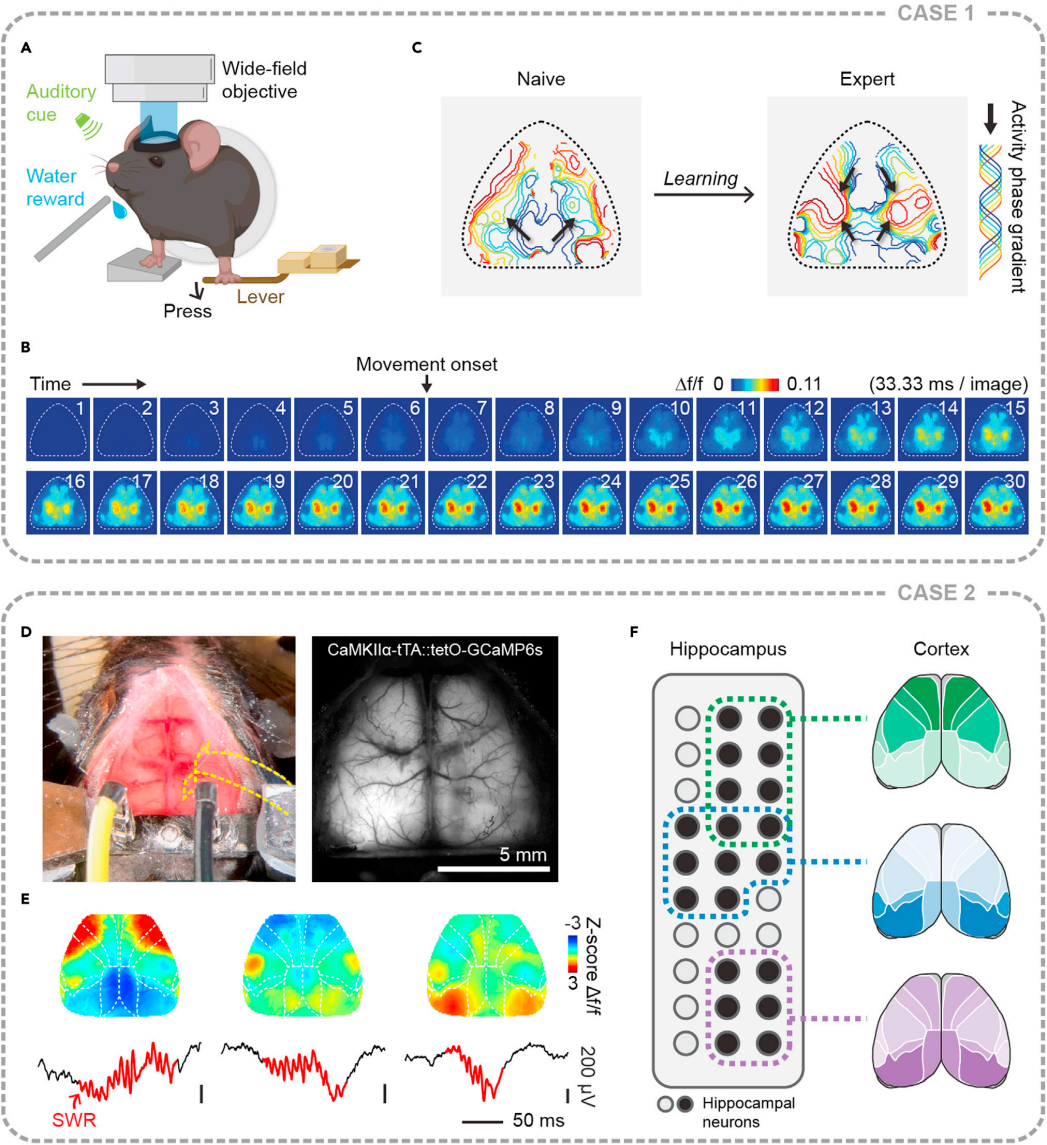
Two example cases using wide-field calcium imaging to investigate cortex-wide dynamics (A) Imaging cortex-wide activity with wide-field calcium imaging during a motor learning task. In this task, water-restricted mice are trained to press a lever during an auditory cue to receive a water reward. (B) Cortex-wide activity aligned to the lever-pressing movement onset averaged across movements in a single session from an example animal. Note that the lever-pressing movement evokes distributed activation of most of the cortex. (C) Learning changes the activity flow throughout the cortex, inducing a secondary activity stream flowing from the secondary motor cortex to the rest of the cortex. Black arrows indicate the directions of the activity flow. Adapted from Makino et al. (2017). (D) Simultaneous recordings of cortex-wide activity using wide-field calcium imaging and hippocampal activity using a newly developed transparent and flexible electrode array (Neuro-FITM). Yellow dashed lines mark the edge of the electrode array for visualization. Left, wide-field surgical preparation with the electrode array implanted. Right, example field of view under the wide-field microscope. (E) Diverse cortical activity patterns during hippocampal SWRs. Adapted from Liu et al. (2021). (F) Different cortex-wide activity patterns are associated with distinct hippocampal neural population activity during SWRs. Scale bars in (D), 5 mm, in (E), vertical, 200 μV, horizontal, 50 ms.

This study investigated how motor learning modulates the cortex-wide neural dynamics during the acquisition of new motor skills. We performed longitudinal wide-field calcium imaging in transgenic mice expressing GCaMP6s in cortical excitatory neurons while they learned a lever-press motor task (Figure 5A). By analyzing the spatiotemporal properties of the cortex-wide activity during lever-pressing movements (Figure 5B), we found that a more compressed and reliable cortex-wide activity gradually emerged with learning. Furthermore, learning also rerouted the cortical activity flow, inducing a novel activity stream flowing from the secondary motor cortex (M2) to the rest of the cortex (Figure 5C). Meanwhile, M2 acquired an indispensable role in coordinating cortex-wide dynamics for newly acquired motor skills. These results suggest that motor learning reconfigures the cortex-wide neural network, forming a more efficient and reliable representation of the learned behavior.

### Case 2: Characterizing cortex-wide activity associated with hippocampal sharp-wave ripples (SWRs) (Liu et al., 2021)

This study investigated the large-scale interactions between cortex and hippocampus during hippocampal SWRs during spontaneous activity in awake mice. We performed simultaneous recordings of cortex-wide activity using wide-field calcium imaging and hippocampal activity using a newly developed transparent and flexible electrode array (Neuro-FITM, Figure 5D). We found diverse cortical activity patterns accompanied hippocampal SWRs (Figure 5E). Furthermore, using dimensionality reduction analysis, we extracted several major types of cortex-wide activity patterns during SWRs, each associated with distinct hippocampal neural population activity (Figure 5F). These results suggest selective and diverse interactions between the large-scale cortical network and hippocampus during SWRs.

## Quantification and statistical analysis

**Timing: ∼24 h/1-h continuous imaging at 30 Hz**

### Obtain Δf/f time series for each pixel in wide-field images

This section describes the steps to obtain Δf/f from image frames. Data processing is performed using custom codes in MATLAB (GitHub: https://github.com/CRen2333/Wide-field-calcium-imaging).

1. To reduce data size and noise, images can first be binned from 512 × 512 pixels (∼21.5 × 21.5 μm^2^/pixel) to 128 × 128 pixels (∼86 × 86 μm^2^/pixel).

***Note:*** Although the binning sacrifices the spatial resolution, it also reduces the noise and compresses the data size for processing. After binning, the spatial resolution (∼86 × 86 μm^2^/pixel) is still sufficient to resolve activity within individual cortical regions (Figure 4B).

**Figure 6:**
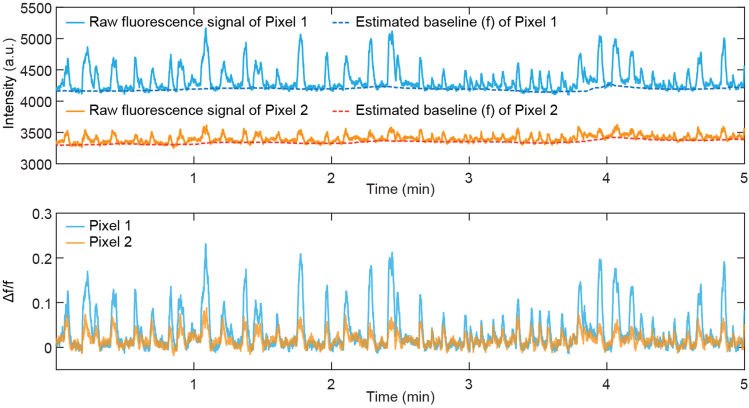
Obtain Δf/f time series for each pixel in wide-field images Raw fluorescence signal, estimated baseline (f), and Δf/f traces of two example pixels with different brightness are shown here. Note that the estimated baseline (f) captures the difference in fluorescence intensity between two pixels and the slow drifts in raw fluorescence traces (potentially due to the slow decay time of GCaMP6s). After normalization, the Δf/f time series shows a more stable baseline level while fast transients are retained.

***Note:*** The estimated baseline generated by this parameter set is close to the fluorescence intensity where no apparent activation occurs. It filters out fast signal transients but captures slow drifts in the fluorescence trace (e.g., the gradual decay of signal intensity during long-time imaging or accumulated intensity drifts due to the slow decay time of GCaMP6s). After normalization, the Δf/f time series shows a more stable baseline level while fast transients are retained.

2. To account for different fluorescence intensities across pixels, Δf/f time series for each pixel is calculated to measure the relative changes in fluorescence intensity caused by activity (Figure 6).
  a. The time-varying baseline fluorescence (f) of each pixel is estimated for a given time point as the 10th percentile value over 30 s around it.
  b. For the beginning and end of each continuous image stack, the following and preceding 15 s window is used to determine the baseline, respectively.

### Image registration across sessions within the same mouse

This section describes the steps to register image frames across multiple imaging sessions within each animal.

***Note:*** We recommend doing image registration before running principal component analysis (PCA) and independent component analysis (ICA) on imaging data combined across sessions as described below. Registration can minimize the effects of variability in head position across sessions so that the top components from PCA and ICA will be focused on variances from brain activity.

3. Calculate the average frame of each imaging session for registration, and the average frame of the first imaging session is taken as the reference image.
4. For each imaging session, we use ‘imregconfig’ and ‘imregtform’ MATLAB functions to generate a geometric transformation matrix of the averaged frame to align with the reference image.
5. Use ‘imwarp’ MATLAB function to apply the geometric transformation matrix to the entire imaging session.

### Removing hemodynamic artifacts using principal component analysis and independent component analysis

**Figure 7:**
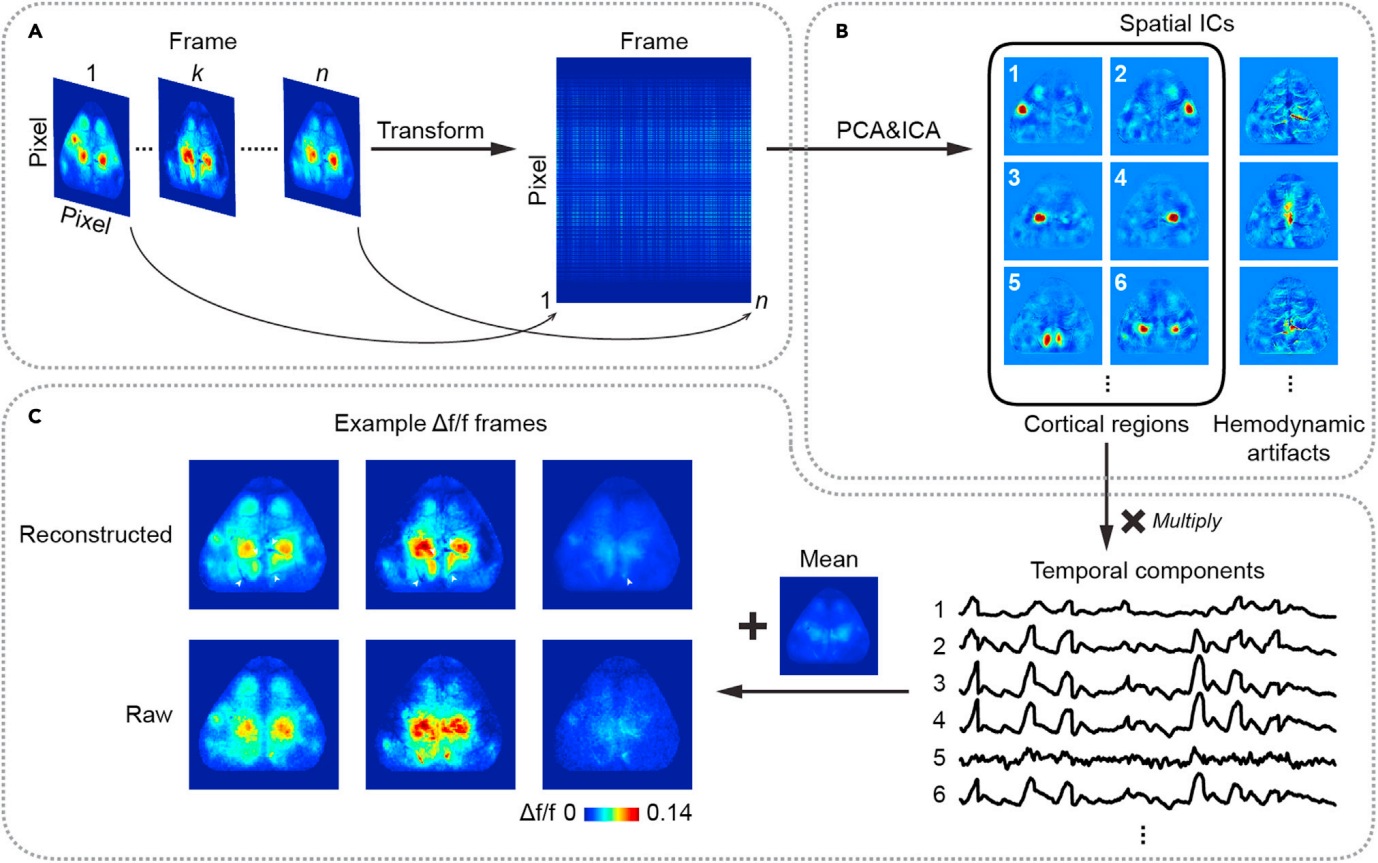
Procedures of using PCA-and-ICA analysis to remove hemodynamic artifacts from wide-field signals (A) Transform the 3D Δf/f image stack (row pixel × column pixel × frame) to a 2D matrix (pixel × frame) for PCA where pixels are treated as variables and frames are treated as observations. (B) Extract spatial components by PCA-and-ICA analysis. Most spatial ICs correspond to known cortical regions or hemodynamic artifacts. ICs corresponding to artifacts are visually identified and excluded. (C) Reconstruct Δf/f images by multiplying the remaining ICs corresponding to cortical regions with their time series, then adding the mean value from the original Δf/f image stack for each pixel. The resulting reconstructed images retain the activity of cortical regions while effectively reducing artifacts. White arrowheads indicate the elimination of hemodynamic signals in reconstructed images compared to the raw images.

The excitation and emission wavelengths (∼485 and ∼520 nm, respectively) of GCaMP are in the absorption spectrum of oxyhemoglobin and deoxyhemoglobin, so the raw fluorescence signal of wide-field calcium imaging is contaminated by hemodynamic changes. We use a PCA-and-ICA-based method to remove hemodynamic signals from wide-field signals (Figure 7). In brief, we first perform PCA to reduce the data dimensionality while still preserving most of the variance. Next, different signal sources (independent components, ICs) are extracted from principal components (PCs) based on their statistical independence through ICA. Identified ICs correspond to functionally distinct cortical regions and artifacts.

***Note:*** This strategy of identifying independent signal sources in recorded mixtures has been widely used for analyzing data of other recording modalities, such as functional magnetic resonance imaging (fMRI) and electroencephalogram (EEG) (Brown et al., 2001). It is also simple to implement and effective for data from wide-field calcium imaging.

***Note:*** Extracting 40 ICs from 37.8 GB data (128 × 128 pixels × 324,000 frames of a 3-h imaging session) takes ∼15–20 h using JADER algorithm for ICA (including ∼1 h for PCA) on a server with dual CPU (Intel Xeon E5-2630 v4, 6 cores and 12 threads each). Different ICA algorithms can also be applied based on the available computing power, see reference (Sahonero-Alvarez and Calderon, 2017) for a detailed comparison between different algorithms. However, we also note that hemodynamics signals can be removed by using a secondary wavelength of light at the isosbestic point of the sensor to estimate the reflectance changes caused by hemodynamics (Ma et al., 2016).

6. First, transform the 3D Δf/f image stack (row pixel × column pixel × frame) to a 2D matrix (pixel × frame) for PCA (Figure 7A).
7. Extract spatial PCs for ICA. In PCA, we treat pixels as variables and frames as observations. The first 40 PCs that explain ∼95% of the total variance are retained for subsequent ICA. For GCaMP signals from cortex-wide excitatory neurons, 30–50 PCs are often sufficient to capture > 90% of the total variance.

***Note:*** In studies using different indicators or imaging different neuronal populations (Musall et al., 2019; Shimaoka et al., 2019), the number of retained PCs may vary based on the signal quality and the fraction of variance explained.

8. Feed retained PCs into ICA to extract functional modules. The ICA algorithm adopted in our protocol is JADER, which decomposes mixed signals into independent components by minimizing the mutual information (Cardoso, 1999). JADER extracts 40 ICs based on the first 40 PCs. Here, ICs are spatial modes forced to be as independent as possible. Most ICs correspond to known cortical regions or hemodynamic artifacts (Figure 7B).
9. ICs corresponding to artifacts are visually identified and excluded.

***Note:*** The number of components excluded can vary across animals due to individual variations in blood vessel pattern and hemodynamic signal strength.

10. Reconstruct images using the remaining ICs corresponding to cortical regions by calculating the products of these ICs with their time series (Figure 7C).
11. For each pixel, its mean value from the original Δf/f image stack is added, because the initial PCA subtracts the mean of each pixel. The resulting reconstructed image stacks specifically retain the activity of cortical regions while effectively reducing artifacts (Figure 7C).

## Limitations

First, the skull-intact surgical preparation requires relatively strong fluorescence signals in imaging samples due to limited skull transmission (Soleimanzad et al., 2017). To image dim samples (e.g., GCaMP fluorescence signals from sparse neuronal populations), replacing the skull with a transparent glass window (Kim et al., 2016) to increase light transmission might be necessary to improve the signal-to-noise ratio. Second, due to the light scattering in brain tissue, the wide-field signals mainly come from superficial cortical layers. Therefore, it may be impracticable to image deep layers with wide-field calcium imaging directly. Third, as wide-field calcium imaging does not possess single-cell resolution, the signal in each pixel is an integration of activity from both local neurons and long-range axonal projections. Therefore, the wide-field calcium signal is not a clean representation of local neural activity, which calls for caution in interpreting the results.

Recently, wide-field calcium imaging has been combined with other recording modalities, such as fMRI, two-photon calcium imaging, and electrophysiological recordings (Barson et al., 2020; Clancy et al., 2019; Lake et al., 2020; Liu et al., 2021; Peters et al., 2021; Xiao et al., 2017). These multimodal recording techniques complement the limitations of wide-field calcium imaging in recording depth and spatial resolution, extending the applications in at least two aspects. First, they offer new opportunities to investigate the large-scale interactions between the cortex and subcortical regions during various cognitive processes. Second, simultaneous recoding at single-cell and cortex-wide level bridges the gap between neural activities at different spatial scales and helps to reveal how local circuits are embedded in larger neural networks.

## Troubleshooting

### Problem 1

Bloodstains on the skull surface and in glue layers (related to step 6).

### Potential solution

Before applying cyanoacrylate glue, use a dry cotton swab to press the bleeding spot until bleeding stops and rinse the skull with saline several times to clean any bloodstains. If the bleeding does not stop after pressing for 10 min, terminate the surgery and exclude the animal from the experiment. If bleeding starts after applying cyanoacrylate glue and spreads, carefully drill off the glue with a dental drill after the glue layer cures. To avoid overheating during drilling, stop every 30 s to let the surface cool down and blow with a compressed air duster to facilitate cooling. Next, blow off the debris with a compressed air duster and reapply cyanoacrylate glue to form a smooth and transparent layer. If bleeding does not stop or the bloodstains contaminate a large area (e.g., covering more than half of a cortical region), terminate the surgery and exclude the animal from the experiment.

### Problem 2

Glue layers are opaque (related to steps 8–9).

### Potential solution

Make sure the soft tissues are cleared, and the skull is dry before applying cyanoacrylate glue. The skull should become whiter and less transparent as it dries (see Figure 3E for the comparison between dry and moisturized skulls). To ensure dryness, gently blow the skull with a compressed air duster to facilitate drying until the skull stops becoming further whiter. However, do not blow too hard as a strong airflow may detach the skin around the cuts and cause bleeding near the retro-orbital sinus.

### Problem 3

Infection along the scalp cuts (related to step 10).

### Potential solution

The infection will loosen the attachment between glue layers and the skull. Add cyanoacrylate glue to seal the cuts further. Inject Baytril (10 mg/kg of body weight) subcutaneously for consecutive 3–5 days to stop infection. If the infection continues, terminate the experiment and exclude the animal from the study.

### Problem 4

Headbar detaches during head-fixation (related to step 12).

### Potential solution

This happens when there is not enough cyanoacrylate glue and dental acrylic cement between bones and the headbar. If only a small gap appears between the skull and glue layer, fill the gap and seal the edge with cyanoacrylate glue. Next, add cyanoacrylate glue and dental acrylic cement between bones and the headbar to form a firm fixation. Inject Baytril (10 mg/kg of body weight) subcutaneously to prevent infection. If the detaching cannot be fixed or it causes excessive bleeding on the skull, terminate the experiment and exclude the animal from the study.

### Problem 5

The surface of glue layers is wrinkled and frosted (related to steps 13–14).

### Potential solution

This happens when a large amount of cyanoacrylate glue is applied at a time, and the curing speed differs between the center and the edge. Only apply a small amount of glue each time and let the glue cure before adding another layer. If this issue still occurs, carefully shave the glue to an even layer with a dental drill. To avoid overheating during drilling, stop every 30 s to let the surface cool down and blow with a compressed air duster to facilitate cooling. After shaving, blow off debris with a compressed air duster and reapply glue to form a smooth and transparent surface.

## Resource availability

### Lead contact

Further information and requests for resources and reagents should be directed to and will be fulfilled by the lead contact, Takaki Komiyama (tkomiyama@ucsd.edu).

### Materials availability

This study did not generate new materials.

## Data Availability

Data are available from the corresponding author upon reasonable request. The custom code for data processing is available at GitHub (https://github.com/CRen2333/Wide-field-calcium-imaging).

## References

[bib1] Allen W.E., Kauvar I.V., Chen M.Z., Richman E.B., Yang S.J., Chan K., Gradinaru V., Deverman B.E., Luo L., Deisseroth K. (2017). Global representations of goal-directed behavior in distinct cell types of mouse neocortex. Neuron.

[bib2] Barson D., Hamodi A.S., Shen X., Lur G., Constable R.T., Cardin J.A., Crair M.C., Higley M.J. (2020). Simultaneous mesoscopic and two-photon imaging of neuronal activity in cortical circuits. Nat. Methods.

[bib3] Brown G.D., Yamada S., Sejnowski T.J. (2001). Independent component analysis at the neural cocktail party. Trends Neurosci..

[bib4] Cardin J.A., Crair M.C., Higley M.J. (2020). Mesoscopic imaging: shining a wide light on large-scale neural dynamics. Neuron.

[bib5] Cardoso J.F. (1999). High-order contrasts for independent component analysis. Neural Comput..

[bib6] Chan K.Y., Jang M.J., Yoo B.B., Greenbaum A., Ravi N., Wu W., Sánchez-guardado L., Lois C., Mazmanian S.K., Deverman B.E. (2017). Engineered adeno-associated viruses for efficient and noninvasive gene delivery throughout the central and peripheral nervous systems. Nat. Neurosci..

[bib7] Clancy K.B., Orsolic I., Mrsic-Flogel T.D. (2019). Locomotion-dependent remapping of distributed cortical networks. Nat. Neurosci..

[bib8] Couto J., Musall S., Sun X.R., Khanal A., Gluf S., Saxena S., Kinsella I., Abe T., Cunningham J.P., Paninski L. (2021). Chronic, cortex-wide imaging of specific cell populations during behavior. Nat. Protoc..

[bib9] Gilad A., Gallero-Salas Y., Groos D., Helmchen F. (2018). Behavioral strategy determines frontal or posterior location of short-term memory in neocortex. Neuron.

[bib10] Kim T.H., Zhang Y., Lecoq J., Jung J.C., Li J., Zeng H., Niell C.M., Schnitzer M.J. (2016). Long-term optical access to an estimated one million neurons in the live mouse cortex. Cell Rep..

[bib11] Lake E.M.R., Ge X., Shen X., Herman P., Hyder F., Cardin J.A., Higley M.J., Scheinost D., Papademetris X., Crair M.C. (2020). Simultaneous cortex-wide fluorescence Ca^2+^ imaging and whole-brain fMRI. Nat. Methods.

[bib12] Liu X., Ren C., Lu Y., Liu Y., Kim J.H., Leutgeb S., Komiyama T., Kuzum D. (2021). Multimodal neural recordings with Neuro-FITM uncover diverse patterns of cortical–hippocampal interactions. Nat. Neurosci..

[bib13] Lohani S., Moberly A.H., Benisty H., Landa B., Jing M., Li Y., Higley M.J., Cardin J.A. (2020). Dual color mesoscopic imaging reveals spatiotemporally heterogeneous coordination of cholinergic and neocortical activity. BioRxiv.

[bib14] Ma Y., Shaik M., Kim S., Kozberg M., Zhao H., Yu H., Hillman E. (2016). Wide-field optical mapping of neural activity and brain haemodynamics: considerations and novel approaches. Philos. Trans. R. Soc. Lond. B. Biol. Sci..

[bib15] Makino H., Ren C., Liu H., Kim A.N., Kondapaneni N., Liu X., Kuzum D., Komiyama T. (2017). Transformation of cortex-wide emergent properties during motor learning. Neuron.

[bib16] Mayford M., Bach M.E., Huang Y.Y., Wang L., Hawkins R.D., Kandel E.R. (1996). Control of memory formation through regulated expression of a CaMKII transgene. Science.

[bib17] Michelson N.J., Vanni M.P., Murphy T.H. (2019). Comparison between transgenic and AAV-PHP.eB-mediated expression of GCaMP6s using in vivo wide-field functional imaging of brain activity. Neurophotonics.

[bib18] Musall S., Kaufman M.T., Juavinett A.L., Gluf S., Churchland A.K. (2019). Single-trial neural dynamics are dominated by richly varied movements. Nat. Neurosci..

[bib19] Nakamura M., Yako T., Kuse Y., Inoue Y., Nishinaka A., Nakamura S., Shimazawa M., Hara H. (2018). Exposure to excessive blue LED light damages retinal pigment epithelium and photoreceptors of pigmented mice. Exp. Eye Res..

[bib20] Peters A.J., Fabre J.M.J., Steinmetz N.A., Harris K.D., Carandini M. (2021). Striatal activity topographically reflects cortical activity. Nature.

[bib21] Ren C., Komiyama T. (2021). Characterizing cortex-wide dynamics with wide-field calcium imaging. J. Neurosci..

[bib22] Sahonero-Alvarez G., Calderon H. (2017). IMCIC 2017 - 8th Int. Multi-conference Complexity, Informatics Cybern. Proc. 2017-march.

[bib23] Shimaoka D., Steinmetz N.A., Harris K.D., Carandini M. (2019). The impact of bilateral ongoing activity on evoked responses in mouse cortex. Elife.

[bib24] Soleimanzad H., Gurden H., Pain F. (2017). Optical properties of mice skull bone in the 455- to 705-nm range. J. Biomed. Opt..

[bib25] Valley M.T., Moore M.G., Zhuang J., Mesa N., Castelli D., Sullivan D., Reimers M., Waters J. (2020). Separation of hemodynamic signals from GCaMP fluorescence measured with wide-field imaging. J. Neurophysiol..

[bib26] Wang Q., Ding S.L., Li Y., Royall J., Feng D., Lesnar P., Graddis N., Naeemi M., Facer B., Ho A. (2020). The allen mouse brain common coordinate framework: a 3D reference atlas. Cell.

[bib27] Wekselblatt J.B., Flister E.D., Piscopo D.M., Niell C.M. (2016). Large-scale imaging of cortical dynamics during sensory perception and behavior. J. Neurophysiol..

[bib28] Xiao D., Vanni M.P., Mitelut C.C., Chan A.W., LeDue J.M., Xie Y., Chen A.C., Swindale N.V., Murphy T.H. (2017). Mapping cortical mesoscopic networks of single spiking cortical or sub-cortical neurons. Elife.

[bib29] Xie Y., Chan A.W., McGirr A., Xue S., Xiao D., Zeng H., Murphy T.H. (2016). Resolution of high-frequency mesoscale intracortical maps using the genetically encoded glutamate sensor iGluSnFR. J. Neurosci..

[bib30] Yizhar O., Fenno L.E., Davidson T.J., Mogri M., Deisseroth K. (2011). Optogenetics in neural systems. Neuron.

